# Legal Liabilities of Physicians and Surgeons

**Published:** 1866

**Authors:** 


### Editorial Department.

### Legal Liabilities of Physicians and Surgeons.

The vexatious suit for mal-practice recently submitted to our Court, calls our attention to this subject at the present time, and induces a consideration of some of the reasons why physicians and surgeons, the latter especially, are not as safe with courts and jury as are other persons, not belonging to the professions or to corporations. That surgeons are, and of right should be, liable for ignorance or unskilful management in the treatment of cases entrusted to their care, there can be no doubt, and there is no reason for complaint that such cases should be submitted to competent and impartial investigation. It is generally understood by physicians that the sympathy of a jury of citizens is not generally with the doctor, but rather on the side of the poor, ill-advised, unfortunate victim of iucurable injury, so that often when no real blame can be attached to the medical attendant he may be obliged to help pay the costs of a litigation, which every one instrumental in encouraging, of right, ought to bear alone.

It is ignorantly supposed by the great majority of people that deformity and disability after injury constitutes ground of complaint, and that the surgeon is under obligation to make everything perfect which he attempts to remedy. A jury when told that oblique fracture of bones always results in shortening, do not understand that this fact constitutes perfect exemption of the surgeon from liability, but still hold him responsible for a result which human ingenuity has never yet devised means to prevent. When it is shown by unanimous testimony upon both sides that stiffness of joints results from inflammation, effusions and long continued rest—from causes wholly distinct from treatment, and beyond the control of a surgeon, a jury will not be able to see that there is no mal-practice, but that on the contrary the very best possible attention has been bestowed. Men uneducated to the distinction, are often unable to see the value of a *principle*, and are ready to compromise what they see clearly to be right, to the prejudice or caprice of an associate who thinks, perhaps innocently, that a few dollars are of no great account in comparison with the loss and pain sustained by serious injury, added to litigation, even though it be ill-advised and unjustifiable.

In the case which we have published somewhat in detail, on account of a local interest in its results, the verdict of the jury is certainly explainable upon the basis indicated, and upon no other. It appears that mal-practice was not entertained at all, for certainly fifty dollars, without costs, was a sum too insignificant to justify such conclusion. The medical testimony as furnished us by counsel for defense, is in some respects quite remarkable. One physician called for plaintiff, who himself uses crooked splint, testifies truly that there is nothing in the condition of the arm, showing improper treatment—that such results might follow the best directed efforts. One testifies that straight splint is *improper*, etc., etc., but he has never heard of Colles' Fracture, and says that Hamilton's work on Fractures and Dislocations "is authority with *some* surgeons." Reads Gross' Surgery every week, but knows of no author who recommends straight splints. The other medical testimony is direct, positive and harmonious; it attributes disability wholly to injury, and justifies the use of straight splints in cases of fracture of the lower end of radius; shows that as much stiffness and deformity may and often does follow slighter injuries or similar injury after the best treatment.

The case in itself is of very little importance, and the verdict of the jury when rightly understood is decidedly for the defendant. While it exonerates him from the charge of mal-practice, it still allows a mere trifle to a patient who had been made to believe herself improperly treated, because the use of the hand was impaired. She had come to believe this, however, upon very insufficient and uncertain grounds, and one eminent physician who was early consulted without mistrusting that his opinions were wanted for any other than professional purposes, congratulated her upon the favorable results she had obtained after so severe an injury, and told her she might be "indeed thankful for so good a hand." This fact did not appear in evidence, but the next hearing will produce it. Dr. ——— was not invited to give evidence for plaintiff.

It is a humiliating thing, that there should be any discrepancy in medical testimony in such a case. There was an almost unanimous agreement upon every point submitted, and it seems incredible that there could have been any disagreement whatever. The principle involved is of some importance, not only to the profession but also to the public.

Suits for mal-practice in Buffalo have grown so rare, that they would be unknown unless imported, as in this instance. A few years since a prominent and wealthy physician in the city defended a suit of this sort successfully, contributing something over one thousand dollars in his own defence; in defence of his profession, and the community untold thousands; for the community are defended and benefited when justice and right are maintained and established; the profession also is strengthened when its true duties and liabilities are rendered apparent. The influence of this suit has been felt by the community for a long time, and there has hardly since appeared a physician willing to prejudice a jury, without just grounds.

The real liability of physicians and surgeons is much better understood now than formerly. Deformities after fractures, by Frank Hastings Hamilton, revolutionized the opinions of surgeons the world over, and a work by the same author upon Fractures and Dislocations, is "authority" in that department wherever men read the English language. If there are physicians in America who have never heard of the distinguished surgeon and author of Dublin, Mr. Colles, who in 1814 gave a very full and correct account of fracture of the lower end of radius, we venture the opinion that there is not a surgeon of any attainment in all Europe, who has not read or heard of Hamilton's surgery, and regards its teachings as eminently correct and reliable. We gladly make a little digression to mention the name of an author who has done so much for surgery as Frank H. Hamilton, and who, at the same time, has given a standard authority upon deformities after fractures, which will influence courts and juries, and constitute an imperishable defence and refuge, making his name and fame immortal.

It is folly to complain of the prejudice or caprice often manifested by a jury in suits for mal-practice. It cannot be expected that all its members can discriminate where men supposed to be educated to the point, disagree. The profession must be first elevated to a degree of intelligence sufficient to insure harmony in the truth, and *truth* must be the standard of trial. Claims of malpractice most frequently grow out of want of fidelity in the profession, and can only be suppressed and avoided when practitioners observe the *Golden Rule*, "Therefore, all things whatsoever ye would that men should do to you, do ye even so to them."

